# Inhibition of coronavirus HCoV-OC43 by targeting the eIF4F complex

**DOI:** 10.3389/fphar.2022.1029093

**Published:** 2022-12-01

**Authors:** Yongmei Feng, Stefan Grotegut, Predrag Jovanovic, Valentina Gandin, Steven H. Olson, Rabi Murad, Anne Beall, Sharon Colayco, Paul De-Jesus, Sumit Chanda, Brian P. English, Robert H. Singer, Michael Jackson, Ivan Topisirovic, Ze’ev A. Ronai

**Affiliations:** ^1^ Cancer Center at Sanford Burnham Prebys Medical Discovery Institute, La Jolla, CA, United States; ^2^ Conrad Prebys Center for Chemical Genomics at Sanford Burnham Prebys Medical Discovery Institute, La Jolla, CA, United States; ^3^ Lady Davis Institute, SMBD Jewish General Hospital, Gerald Bronfman Department of Oncology and Division of Experimental Medicine, McGill University, Montreal, QC, Canada; ^4^ Janelia Research Campus, Howard Hughes Medical Institute, Ashburn, VA, United States; ^5^ Immunology and Infectious Disease Center at Sanford Burnham Prebys Medical Discovery Institute, La Jolla, CA, United States

**Keywords:** COVID-19, OC43, SARS-CoV-2, coronavirus, eIF4F, translation initiation complex, Vero E6, A549

## Abstract

The translation initiation complex 4F (eIF4F) is a rate-limiting factor in protein synthesis. Alterations in eIF4F activity are linked to several diseases, including cancer and infectious diseases. To this end, coronaviruses require eIF4F complex activity to produce proteins essential for their life cycle. Efforts to target coronaviruses by abrogating translation have been largely limited to repurposing existing eIF4F complex inhibitors. Here, we report the results of a high throughput screen to identify small molecules that disrupt eIF4F complex formation and inhibit coronavirus RNA and protein levels. Of 338,000 small molecules screened for inhibition of the eIF4F-driven, CAP-dependent translation, we identified SBI-1232 and two structurally related analogs, SBI-5844 and SBI-0498, that inhibit human coronavirus OC43 (HCoV-OC43; OC43) with minimal cell toxicity. Notably, gene expression changes after OC43 infection of Vero E6 or A549 cells were effectively reverted upon treatment with SBI-5844 or SBI-0498. Moreover, SBI-5844 or SBI-0498 treatment effectively impeded the eIF4F complex assembly, with concomitant inhibition of newly synthesized OC43 nucleocapsid protein and OC43 RNA and protein levels. Overall, we identify SBI-5844 and SBI-0498 as small molecules targeting the eIF4F complex that may limit coronavirus transcripts and proteins, thereby representing a basis for developing novel therapeutic modalities against coronaviruses.

## Highlights

• SBI-5844 and SBI-0498 are novel compounds that disrupt the eIF4F complex.
• SBI-5844 and SBI-0498 inhibit HCoV-OC43 with minimal toxicity in cell culture.
• SBI-5844 and SBI-0498 reverse gene expression signatures induced upon HCoV-OC43 infection.
• Comparable gene expression signatures were seen following HCoV-OC43 and SARS-CoV-2 infection.

## Introduction

The severe acute respiratory syndrome coronavirus (SARS-CoV) is one of three coronaviruses introduced into the human population over the past 2 decades, all causing substantial morbidity and mortality (Hu et al., 2021; Yesudhas et al., 2021). Outcomes associated with Middle East respiratory syndrome coronavirus (MERS-CoV) and the COVID-19 pandemic caused by the SARS-CoV-2 virus resemble earlier coronavirus epidemics caused by zoonotic transmission (Chen et al., 2021; Shahrajabian et al., 2021). Both SARS and MERS-like coronaviruses were detected in bat reservoirs and shown to replicate in cultured human lung cells (Singh and Yi, 2021; Zhang et al., 2021). These events reinforce the urgent need to identify effective anti-viral therapeutics to combat current and future pandemics.

SARS-CoV-2 causes a potentially severe respiratory illness with mortality seen in 1%–10% (Meyerowitz-Katz and Merone, 2020; Shoaib et al., 2021) of the aged population (O'Driscoll et al., 2021). Several compounds that either modulate the human immune response or have anti-inflammatory effects have been evaluated in clinical trials, among which Paxlovid was recently approved by the FDA as an oral anti-viral treatment against SARS-CoV-2 (Sanders et al., 2020; Mahase and Kmietowicz, 2021; Yousefi et al., 2021). Other potential inhibitor targets include host mechanisms required for viral replication, including agents that target host proteases required to induce the fusogenic activity of the SARS-CoV-2 spike protein (Zhu et al., 2020; Peng et al., 2021). Alternative anti-virals have also been explored, including inhibitors of protein glycosylation and folding (Mehta et al., 1997) or the host mRNA translational machinery (McMahon et al., 2011; Dalziel et al., 2014; Heaton et al., 2016; Zhang et al., 2016; Slaine et al., 2017). Along these lines, the inhibitor of the eukaryotic translation initiation factor 4A, silvesterol, reportedly attenuates Ebola viral replication (Biedenkopf et al., 2017). Consistent with the importance of the eIF4F complex for viral replication is the interaction of viral proteins with the translational initiation machinery components, which has been demonstrated for several viruses, including HIV (Jager et al., 2011) and SARS-CoV-2 (Gordon et al., 2020). The eIF4F complex, which integrates mTOR, ERK, and MNK signals, is also required for viral protein synthesis and essential for viral replication (Walsh and Mohr, 2004; Walsh et al., 2005; Perez et al., 2011; Ghasemnejad-Berenji and Pashapour, 2021). eIF4F consists of the cap-binding subunit eIF4E, the scaffolding factor eIF4G, and the DEAD-box RNA helicase eIF4A (Pelletier et al., 2021). The eIF4F complex recruits mRNA to the ribosome and facilitates scanning towards the translation start site (Pelletier et al., 2021). A notable challenge in targeting eIF4F is that it is not only essential for RNA virus protein synthesis and replication, including the SARS-CoV-2 (Cencic et al., 2011; Jan et al., 2016; Montero et al., 2019), but for CAP-dependent translation of most cellular mRNAs (Pelletier and Sonenberg, 2019). Nonetheless, previous research has established that mRNAs encoding housekeeping proteins are dramatically less sensitive to decreases in eIF4F activity compared to transcripts that encode oncogenes and viral proteins (Bhat et al., 2015). Among eIF4A inhibitors that effectively suppress SARS-CoV-2 replication, while causing minimal toxicity to host cells (Bhat et al., 2015; Pelletier et al., 2015), zotatifin is undergoing clinical evaluation (NCT04632381) (Sauvat et al., 2020; Vallianou et al., 2021; Gerson-Gurwitz et al., 2021).

Currently available SARS-CoV-2 treatment modalities include the anti-viral remdesivir (Williamson et al., 2020) and the anti-inflammatory drug dexamethasone, both of which improve patient outcomes in clinical trials and have been approved for emergency use by regulatory agencies (NCT04610541, NCT04707534) (Li et al., 2020; Singh et al., 2020). Notably, remdesivir has limited efficacy (Azevedo et al., 2020; Fatima et al., 2020), while the steroid dexamethasone does not inhibit viral replication directly (Sood et al., 2021; Vecchie et al., 2021), highlighting an unmet clinical need for specific and effective SARS-CoV-2 inhibitors with limited toxicity. Additional SARS-CoV-2 drugs that currently utilized include Olumiant baricitinib (Jorgensen et al., 2020); Sotrovimab (Gupta et al., 2021), Bamlanivimab (Gottlieb et al., 2021) and REGEN-COV Casirivimab and Imdevimab (Weinreich et al., 2021); monoclonal antibodies that help the immune system attack on SARS-CoV-2 by neutralizing the virus ability to enter human cells. Paxlovid is the first oral antiviral drug for COVID-19 (Mahase and Kmietowicz, 2021). Inhibiting the eukaryotic translational machinery could offer numerous advantages over other cellular targets, given the critical dependency of RNA viruses on the host’s translational apparatus.

OC43 (known as beta coronavirus) is among coronaviruses that circulated in the human population in the 1960s and then further evolved to recent versions (HCoV-HKU1 and HCoV-NL63) (Belser et al., 2013). Distinct from severe acute respiratory syndrome coronavirus-2 (SARS-CoV-2), HCoV-OC43 does not lead to respiratory syndromes seen in SARS-CoV-2, although its core single-stranded RNA sequence comparably depends on host components for its successful life cycle.

Here we report a screen to identify putative inhibitors of the eIF4F complex for potential effects on blocking coronavirus RNA and protein levels, using HCoV-OC43 (OC43) infection of Vero E6 cells and the lung epithelial cancer line A549 as models. Of >300,000 small molecules tested, 17 notably inhibited eIF4F *in vitro* and were further assessed for OC43 inhibition and cell toxicity. One of the 17 compounds, and two structurally related analogs, fulfilled these requirements and were further characterized and compared with remdesivir for anti-coronavirus activity in cell culture.

## Results

### Identification of small molecule eIF4F complex inhibitors that block coronavirus RNA and protein levels

**FIGURE 1:**
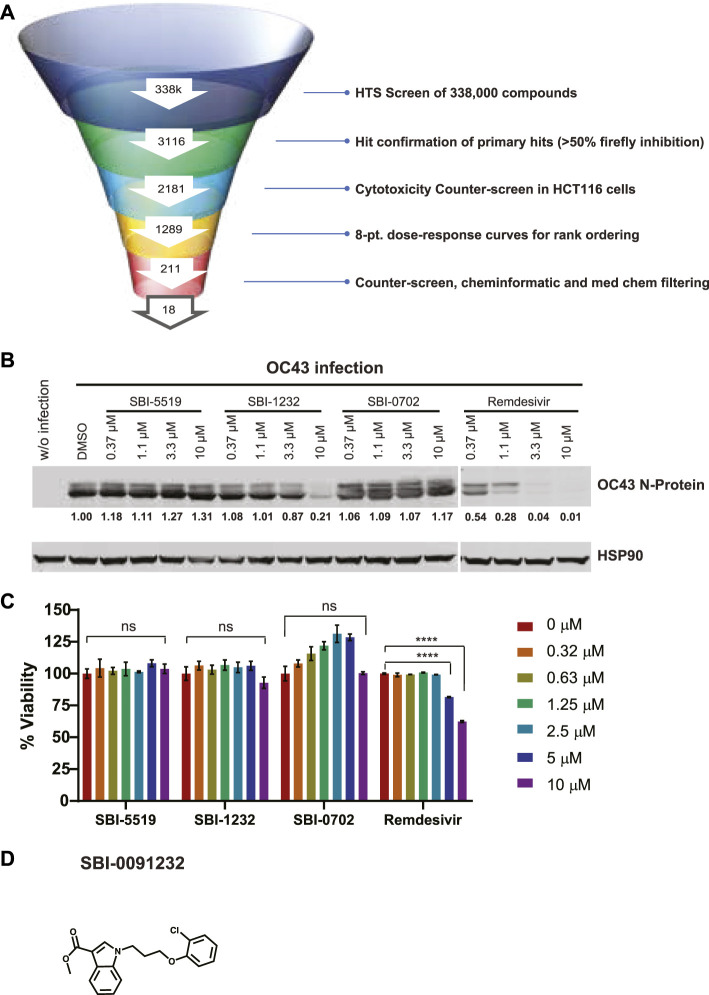
Identification and validation of OC43 N-protein expression inhibitors. **(A)** Testing funnel of high-throughput screening campaign. HTS screen performed using firefly (FF) luciferase readout in RRLs using 338,000 compounds. Hits (3116) were selected for CAP-dependent inhibition based on 50% inhibition of the firefly readout, for CAP-dependent inhibition, and further assessed in a cytotoxicity counter-screen using HCT116 cells, excluding compounds promoting nonspecific inhibition of Ren luciferase activity. The resulting 2,181 compounds were subjected to eight points dose-dependent inhibition assessments for effectiveness, which reduced the number of hits to 221. Counter-screens at 5 and 10 mM and additional chemoinformatic and medicinal chemistry assessment led to selection of 18 compounds. **(B)** Western blot analysis of indicated proteins in A549 cells. Cells (2 x 105) were infected with OC43 and treated immediately following infection with indicated compounds and concentrations for 24 h. Each protein band was quantified by ImageJ, normalized to HSP90 levels as indicated blow blots. **(C)** Viability assay of uninfected A549 cells (8,000 cells/per well) treated with indicated compounds and concentrations. Cell viability was measured 48 h later using CellTiter-Glo. Relative viability was normalized to DMSO-treated control cells. Statistical analysis was performed by one-way ANOVA for the comparison of more than two groups. Data are shown as the mean ± SD, n = 3. *p ≤ 0.05, **p ≤ 0.01, ***p ≤ 0.001, ****p ≤ 0.0001. **(D)** Chemical structure of SBI-0091232.

Considering that translation is critical for RNA virus life-cycle, including the SARS-CoV-2 replication cycle (Cencic et al., 2011; Jan et al., 2016; Montero et al., 2019), we assessed a newly identified set of translation initiation inhibitors for effects on coronavirus replication, using OC43 as a model. To do so, we performed a high-throughput *in vitro* screen for small molecules that effectively inhibit luciferase reporter activity driven by CAP-dependent and -independent mechanisms. This vector encodes for a bicistronic reporter mRNA carrying the encephalomyocarditis virus (EMCV) IRES site between upstream firefly luciferase (fLuc) and downstream renilla Luciferase (rLuc) genes, which is translated in rabbit reticulocyte lysates. A 338,000-compound high-throughput screen (HTS) campaign was performed (Figure 1A and Supplementary Figure S1A) and firefly luciferase activity was monitored as a readout. Using a cut-off of 50% of CAP-dependent luciferase activity, we identified 3,116 primary hits and confirmed 2,181. Among those, 221 compounds exhibited selective CAP-dependent translation inhibition were non-toxic in HCT116 cells and exhibited dose-dependent effects with IC_50_ values <0.1 µM (Figure 1A). Additional assays performed using 5 and 10 µM concentrations excluded the possibility that identified compounds non-specifically inhibit luciferase activity in these *in vitro* based assays. Cheminformatic and medicinal chemistry analyses where then used to better cluster candidate compounds based on structure and drug properties (Figure 1A and Supplementary Figure S1A). Of 18 selected compounds, 17 were commercially available, purchased and assessed for ability to inhibit OC43 replication and potential toxicity. This was achieved by infecting A549 cells with OC43 and subjecting them to treatments with candidate drugs. OC43 replication was assessed using both immunofluorescence (IF) and Western blotting for the OC43 nucleocapsid protein (Supplementary Figures S1B, C), while cell viability was monitored using CellTiter-Glo (Supplementary Figure S1D). As a benchmark for test compound activity, we included remdesivir, a potent SARS-CoV-2 inhibitor (Liang et al., 2020) used clinically (Goldman et al., 2020). SBI-0640756 (SBI-756), a potent inhibitor of the eIF4F complex (Feng et al., 2015), was used as a positive control for the disruption of the eIF4F complex. SBI-756 was not developed further for coronavirus inhibition given its toxicity to cells studied here (Supplementary Figure S1D), although it was used as a reference under conditions minimizing its toxicity (early time points and low concentrations).

Of the 17 compounds, four were selected for further confirmation of OC43 inhibition by Western blotting. That analysis revealed that one compound, SBI-0091232 (SBI-1232), had potency comparable to the positive control SBI-756 in A549 cells (Supplementary Figure S1C). When tested across a dose range in A549 cells, SBI-1232 was effective in decreasing OC43 nucleocapsid protein levels (10 µM), compared with remdesivir (1.1 µM) (Figure 1B). SBI-1232 also exerted minimal toxicity to A549 cells, which was lower than seen with remdesivir, (Figure 1C), leading us to further characterize SBI-1232 (Figure 1D).

### Characterization of selected SBI-1232 analogs for effective inhibition of coronavirus

**FIGURE 2:**
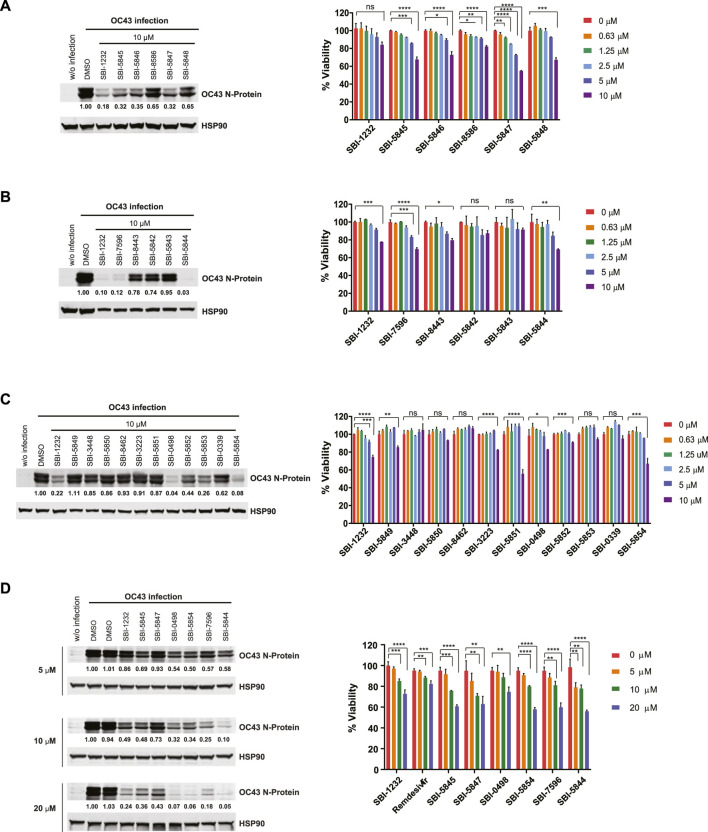
Validation of SBI-1232 analogs for inhibition of OC43 N-protein expression. **(A, B, C and D)** Left, Western blot analysis of indicated proteins from Vero E6 cells (2 x 105) infected with OC43 and treated with indicated compounds for 24 h. Band intensities were quantified against loading control HSP90, as shown below blots. Right panel, viability assay of uninfected Vero E6 cells 48 h after treatment with indicated compounds and concentrations. Statistical analysis was performed by one‐way ANOVA for the comparison of more than two groups. Data are shown as the mean ± SD, n = 3. *p ≤ 0.05, **p ≤ 0.01, ***p ≤ 0.001, ****p ≤ 0.0001.

To improve the ability of SBI-1232 to inhibit viral replication, we selected and obtained 21 analogs that exhibit similar chemical structure from three vendors—ChemBridge (CB), MolPort (MP) and VitaScreen (VS). Selected compounds contained either single point changes or two independent structural changes if a bridging compound was also available. Each compound was assessed for its ability to inhibit OC43 N-protein expression in Vero E6 and A549 cells, lines commonly used to assess coronavirus replication (Plaze et al., 2021; Wang et al., 2021). Comparison of the five CB analogs with SBI-1232 identified two that effectively inhibited OC43 N-protein expression to a degree comparable to SBI-1232 in Vero E6 cells (Figure 2A, left). All three exhibited similar kinetics and equal or greater toxicity, compared with SBI-1232 (Figure 2A, right). Of the five MP analogs, SBI-7596 and SBI-5844 treatment decreased OC43 N-protein expression to greater degree compared with SBI-1232 (Figure 2B, left) although they affected Vero E6 cell viability (Figure 2B, right) at levels comparable to SBI-1232. Among the eleven VS analogs, SBI-0498 and SBI-5854 decreased OC43 N-protein expression more potently than comparable concentrations of SBI-1232 (Figure 2C, left), while exhibiting comparable or somewhat lower toxicity (i.e., SBI-0498) to Vero E6 cells (Figure 2C, right). We next compared the six most potent analogs with lower toxicity from the CB, MP and VS series to SBI-1232 and remdesivir for dose-dependent inhibition of OC43 protein production in both Vero E6 and A549 cells. Among these, SBI-5844 most potently inhibited OC43 N-protein expression in Vero E6 cells (Figure 2D, left). In A549 cells, however, most analogues, including SBI-1232, effectively inhibited viral N-protein expression at lower concentrations, possibly, due to low infection efficiency of A549 relative to Vero E6 cells (Supp. Figure 2A, left). Since our goal was to identify the most effective inhibitor with the least toxicity, we assessed compound effects on viability of A549 and Vero E6 lines. While 10 μM SBI-5844 was more toxic to Vero E6 cells than comparable concentrations of remdesivir (Figure 2D, right), in A549 cells SBI-5844 was less toxic than remdesivir (Supp. Figure 2A, right).

**FIGURE 3:**
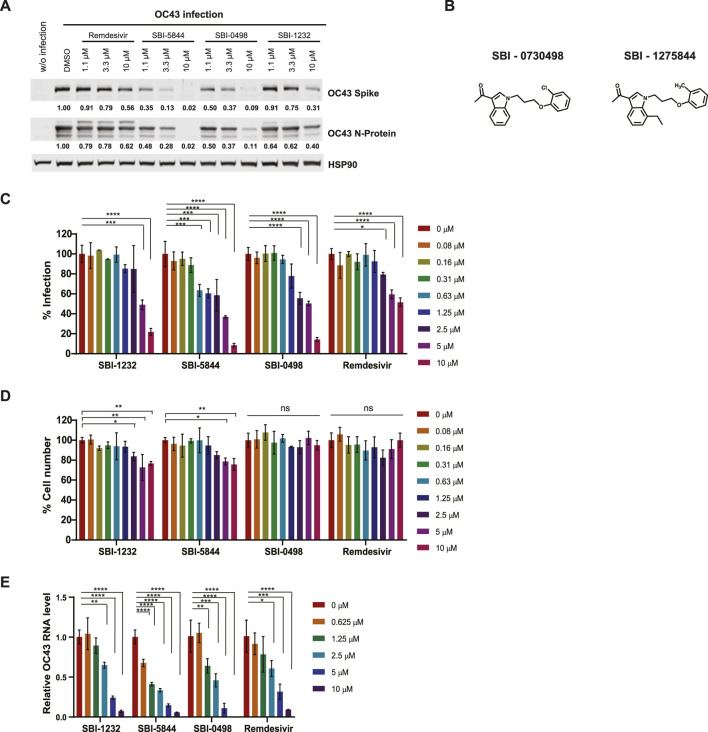
Comparison of virus replication inhibition by selected compounds. **(A)** Vero E6 cells were infected with OC43 and treated with indicated compounds. After 24 h, cells were lysed and subjected to Western blot analysis. Each protein band was quantified by ImageJ, normalized to HSP90 levels as indicated under the blots. **(B)** Chemical structures of SBI-0730498 (SBI-0498) and SBI-1275844 (SBI-5844). **(C)** Vero E6 cells were infected with OC43 and treated with indicated compounds. After 48 h, cells were fixed, and stained with antibodies against the OC43 N-protein and with DAPI for immunofluorescence imaging. Infected cells (positive stained for SARS-CoV-2 N protein) and total cells (DAPI staining) were quantified using Celigo. For each condition, the percent infection was calculated as infected cells/total cells x100, and DMSO-treated control was set to 100% infection. Data shown is the mean ± SD, n = 3. **(D)** Percent cell number was calculated as the compound-treated cell number/DMSO-treated control cell number x100, with DMSO-treated control set as 100%. **(E)** Vero E6 cells were infected with OC43 and treated with indicated compounds. After 24 h, RNA was extracted and subjected to qPCR analysis for virus RNA. OC43 RNA levels are normalized to DMSO treated control cells. Statistical analysis was performed by one‐way ANOVA for the comparison of more than two groups. Data are shown as the mean ± SD, n = 3. *p ≤ 0.05, **p ≤ 0.01, ***p ≤ 0.001, ****p ≤ 0.0001.

To further assess effectiveness of SBI-5844 and SBI-0498 (the top compounds) we compared their effect with that of SBI-1232 and remdesivir on expression of spike and nucleocapsid proteins in OC43-infected Vero E6 cells. Treatment with either compound decreased levels of OC43 spike and nucleocapsid proteins more effectively than did SBI-1232 or remdesivir used at comparable concentrations (Figure 3A). While in A549 cells the effectiveness of SBI-5844 and SBI-0498 was also superior to that of SBI-1232, remdesivir more potently blocked viral protein expression (Supplementary Figure S2B). These findings suggest that SBI-5844 and SBI-0498 (Figure 3B) exhibit activities superior to SBI-1232 and comparable to remdesivir in blocking viral protein expression in cell culture models. When taking the degree of viral infection into consideration, both SBI-5844 and SBI-0498 appear to be superior to remdesivir in Vero E6 cells.

Our Western blot analyses of levels of OC43 spike and nucleocapsid proteins do not assess viral replication *per se*. We used immunofluorescence (IF) analysis of viral particles in OC43-infected Vero E6 cells, in the presence or absence of drug, where we calculated the ratio between fluorescent (infected) and total cells as readout for cells infected by viral particles. In agreement with effects on viral protein levels, treatment with SBI-5844 or SBI-0498 was more potent than treatment with SBI-1232 and remdesivir in decreasing the proportion of infected cells when assessed 48 h post treatment (Figure 3C), while exerting minimal toxicity, as determined by general DAPI staining (Figure 3D). We also quantied levels of OC43 viral RNA in drug-treated *versus* untreated cells. Treatment with SBI-5844 or SBI-0498 for 24 h significantly reduced viral RNA levels relative to control infected cells (Figure 3E). Comparable decreases were seen in A549 cells in terms of proportions of infected cells (Supplementary Figure S2C) and RNA levels (Supplementary Figure S2E), as well as minimal toxicity (Supplementary Figure S2D).

### SBI-5844 or SBI-0498 treatment attenuates eIF4F complex assembly

**FIGURE 4:**
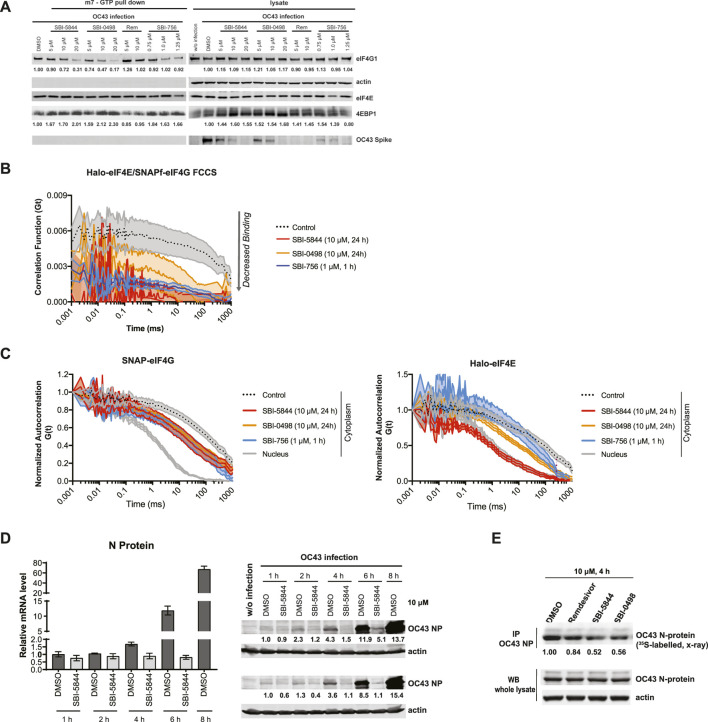
SBI-5844 and SBI-0498 attenuates translation initiation complex function. **(A)** Western blot analysis of indicated proteins prepared from A549 cells infected with OC43 and treated with indicated compounds. Cell lysates were incubated with m7GTP-agarose beads to capture the eIF4F complex. Shown is western blot of both total cell lysates (right panel) and m7GTP-agarose bound proteins (left panel) immunoblotted with indicated antibodies. Each protein band was quantified by ImageJ, normalized to actin (right panel) or eIF4E (left panel) as indicated. **(B)** Simultaneous diffusion of endogenous JF585Halo-eIF4E and JF646SNAPf-eIF4G1 throughout the focal volume was analyzed in living cells by dual color cross-correlation spectroscopy in the indicated conditions (Mean ± SEM; N = 10). Cross-correlation, due to eIF4F:eIF4G interaction, was detected in the cytoplasm of cells treated with vehicle control. Minimal cross-correlation was detected upon treatment with SBI-756, SBI-5844 or SBI-0498. **(C)** Diffusion of endogenous JF646SNAPf-eIF4G1 (left panel) and JF585Halo-eIF4E (right panel) was measured by fluorescence correlation spectroscopy in living cells treated with vehicle control or indicated compounds, in the indicated cellular compartment (Mean ± SEM; N = 10). Nuclear temporal autocorrelation depicts diffusion of free-diffusing JF646SNAPf-eIF4G1 (upper panel) and JF585Halo-eIF4E (lower panel). The autocorrelation curves were normalized for visual comparison of shape. **(D)** Vero E6 cells were infected with OC43 and treated with SBI-5844 for indicated times. RNA and protein were extracted and subjected to RT-qPCR analysis for virus RNA (left, mean ± SD, n = 3) and WB for virus N-protein (right). Each protein band was quantified by ImageJ, normalized to actin levels as indicated in numbers shown under the blots. **(E)** Vero E6 cells were infected with OC43 for 24 h. Cells were then treated with indicated compounds and radiolabeled with 35S-methionineand and 35S-cysteine for newly synthesized protein for 4 h. OC43 N-protein was immunoprecipitated (IP), separated by SDS-PAGE, transferred to PVDF membrane, and exposed to x-ray film. Each band was quantified by ImageJ, normalized to OC43 N-protein level as indicated under the blots.

We next compared top analog candidates with remdesivir for ability to dissociate the eIF4F complex. To do so, we used 7-methyl-GTP (m^7^GTP) sepharose beads to pull down eIF4E and associated eIF4F proteins eIF4G and 4EBP1 in A549 cells, in the presence or absence of the compounds. In this assay, eIF4F complex integrity is reflected by association of eIF4E with eIF4G, while reduced binding of eIF4G to m^7^GTP-bound eIF4E, with concomitant increase in eIF4E:4E-BP1 association, reflects complex disruption (Feng et al., 2015). As anticipated, remdesivir had no ability to dissociate eIF4G from eIF4E in A549 cells, whereas SBI-0498 or SBI-5844 had a notable inhibitory effect comparable to the benchmark inhibitor SBI-756 (Figure 4A) (Feng et al., 2015). The ability of SBI-0498 and SBI-5844 to perturb eIF4F assembly was further assessed in living cells by fluorescence cross-correlation spectroscopy (FCCS) and fluorescence correlation spectroscopy (FCS). In FCS, fluorescence intensity is collected from a detection volume in the order of femtoliter. The technique records fluctuations in the fluorescence signal that occur when thousands of fluorescent molecules move in and out the focal volume. These fluorescence traces are collected over time and are fitted into an autocorrelation function G(τ), that reflects the average residence time of the fluorescent particles in the detection volume (Kim et al., 2007). Dual-color fluorescence cross-correlation spectroscopy detect the time-scale of the diffusion of two different molecules labelled with different fluorophores. Because the fluorescent signal is recorded simultaneously, the cross-correlation is a direct indication of the concomitant movement and so interaction of the two molecules as they transit through the focal volume (Kim et al., 2007). FCS and FCCS allow us to detect in real-time translation initiation events in living cells since it can resolve unbound initiation factors (fast diffusion) or mRNA bound (slow diffusion) (Gandin et al., 2021). Halo- and SNAP_f_-tag suitable for live cell imaging, were fused eIF4E and eIF4G respectively without perturbing their function (Gandin et al., 2021). FCCS analysis detected simultaneous diffusion of endogenously tagged _JF585_Halo-eIF4E and _JF646_SNAPf-eIF4G through the imaged focal volume. Cross-correlation analysis on A549 cells that were subjected to treatment with the top-ranking inhibitors demonstrated that eIF4E and eIF4G interaction was minimal in cells treated with SBI-0498, SBI-5844 or SBI-756 (Figure 4B). Notably, autocorrelations for single _JF646_SNAPf-eIF4G and _JF585_Halo-eIF4E signals demonstrated that their diffusion only marginally slowed in the cytoplasm of cells treated with SBI-756 or SBI-0498 relative to control cells (Figure 4C), suggesting that *in cellula* SBI-756 and SBI-0498 inhibit eIF4E:eIF4G binding without triggering their dissociation from mRNA. On the other hand, autocorrelation obtained in SBI-5844 treated cells, demonstrated that cytoplasmic _JF585_Halo-eIF4E molecules diffuse as fast as their nuclear counterparts (Figure 4C, right panel), mirroring diffusion of free-eIF4E molecules that do not bind the 5′cap (Gandin et al., 2021), suggesting that *in cellula* SBI-5844 can dissociate mRNAs from the eIF4F complex.

### SBI-5844 or SBI-0498 treatment attenuates OC43 protein abundance

Disruption of eIF4F assembly should impact levels of newly synthesized proteins. Given effects of SBI-5844 and SBI-0498 on viral protein expression, and eIF4F complex assembly, we evaluated possible changes in levels of newly synthesized OC43 nucleocapsid protein at early as well as late time points following infection. Time course analysis of OC43 N-protein synthesis was carried out by WB, revealing inhibition of mRNA translation took place early as 1 h after infection, and was more pronounced after 4 h (Figure 4D, right panel), while levels of viral RNA remained unchanged up to the rapid replication phase noted 6 h after infection (Figure 4D, left panel). To further assess possible changes in newly synthesized OC43 nucleocapsid protein 24 h after infection, we performed ^35^S-labeling for pulse chase analysis, whereby newly synthesized proteins are labeled radioactively, allowing to quantify changes in protein abundance. To this end, OC43-infected Vero E6 cells were subjected to ^35^S pulse chase labeling (see Methods) along with treatment with remdesivir, SBI-5844 or SBI-0498 for 4 h. Cell lysates were immunoprecipitated with antibody against OC43 N-protein, separated by SDS-PAGE, transferred to PVDF membrane, and exposed to X-ray film. Quantification of radiolabeled OC43 N-protein revealed a 48% decrease in levels of newly synthesized N-capsid protein in cells subjected to SBI-5844 treatment and a 46% decrease following SBI-0498 treatment, relative to controls (Figure 4E). These observations confirm that both inhibitors impact the eIF4F complex assembly and lower OC43 protein levels, which may explain the concomitant changes in viral transcript and protein levels.

To further assess possible changes in OC43 protein synthesis, following treatment with either SBI-5844 or SBI-0498, we performed polysome profiling. Analysis of Vero E6 cells performed 12 h after OC43 infection followed by the inhibitor treatment revealed general inhibition of translation, based on the decrease in ratio of heavy polysome to monosome fractions (Supplementary Figure S3A). We then assessed possible changes in the translation of viral transcripts in both soluble and endoplasmic reticulum-associated ribosomes. Transcripts for OC43 N protein, RNA-dependent RNA polymerase and 3C-like protease, were quantified using qPCR which was performed in each of the polysome fractions. Notably and unexpectedly, while OC43 mRNAs were localized in fractions corresponding to three or more ribosomes, there was no major difference in their distribution across polysome fractions, comparing control and compound treated cells, while overall abundance of viral RNA was markedly attenuated (Supplementary Figures S3B, S3C, S3D). As expected, host cell histone H3A mRNAs remained associated with heavier polysomes in infected cells and cells treated with compounds, without change in total mRNA abundance (Supplementary Figure S3E).

### Gene expression signatures seen following OC43 infection are rescued by SBI 5844 or SBI-0498 treatment

**FIGURE 5:**
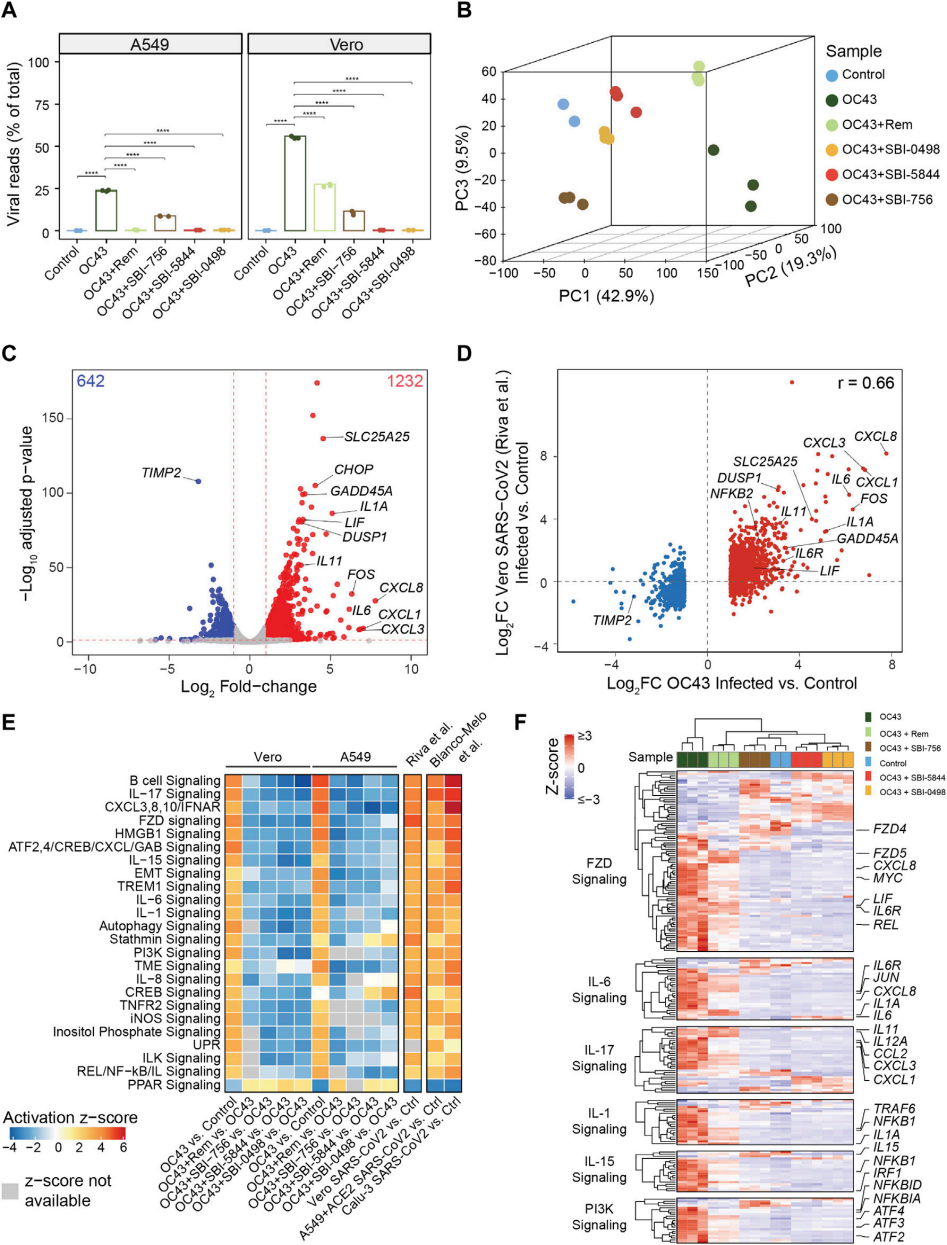
SBI-5844 and SBI-0498 treatment confers transcriptional signatures reflective of uninfected cells to OC43 infected cells. **(A)** RNAseq analysis was performed on RNA extracted from OC43 infected Vero E6 and A549 cells 24 h after treatment with SBI-0498 (10 µM), SBI-5844 (10 µM), SBI-756 (0.5 µM), and remdesivir (5 µM for Vero E6 cells and 1 µM for A549 cells). Viral replication levels in infected, control, and infected + treated samples assessed by the percentage of viral reads relative to total reads. Reads that did not align to human (A549) and African green monkey (Vero) genomes were aligned to OC43 genome. Statistical analysis was performed by one-way ANOVA for the comparison of more than two groups. Data are shown as the mean ± SD, n = 3. *p ≤ 0.05, **p ≤ 0.01, ***p ≤ 0.001, ****p ≤ 0.0001. **(B)** Principal component analysis of Vero E6 samples described in **(A)** PC1, accounting for 42.9% of variance in the data, separates control and infected samples. **(C)** Volcano plot comparing the transcriptomes of OC43 infected and control Vero E6 samples. The numbers of up- and down-regulated genes are indicated on top corners. Representative genes that were up- or down-regulated upon OC43 infection are denoted. **(D)** Comparison of log2 fold-changes of differentially expressed genes in OC43-infected *versus* control uninfected Vero E6 cells in this study with analysis of Vero E6 cells infected with SARS-CoV-256. **(E)** Heatmap depicting Ingenuity Pathway Analysis of pathways representing differentially expressed genes following each of indicated treatments of A549 and Vero E6 cells compared with control cells. Also shown are differentially expressed genes in Vero E656, A549 and Calu-3 cells upon SARS-CoV-2 infection (at low and high MOI for A549) *versus* control cells57. White boxes indicate that activation z-scores are not available. **(F)** Heatmap depicting relative expression of genes related to indicated pathways selected from analysis shown in panel **(E)** relevant to responses to OC43 and treatments in Vero E6. Gene-wise expression z-scores were computed using transcripts per million (TPM) values. Genes (within each pathway) and samples were hierarchically clustered. Selected genes relevant to OC43 infection response are labeled (full scale heat map and gene list are provided in Supplementary Figure S5).

Since SBI-5844 and SBI-0498 analog candidates potently inhibited viral RNA and protein levels and dissociated the eIF4F complex with minimal effects on cell viability, we assessed potential changes in gene expression networks following exposure of OC43-infected A549 or Vero E6 cells to both compounds. To do so, we carried out RNA-seq analysis of both lines after OC43 infection and treatment with either SBI-0498, SBI-5844, SBI-756, or remdesivir. A parallel set of cells subjected to the same treatments were monitored for OC43 protein expression (Supplementary Figure S4A). Notably, viral reads, based on quantitation of RNA viral sequences, indicated 2-fold greater levels of viral RNA in Vero E6 compared to A549 cells, consistent with our Western blotting analysis of OC43 nuclear protein levels. Significantly, SBI-0498 (10 µM), SBI-5844 (10 µM) and SBI-756 (0.5 µM) were more effective in inhibiting OC43 replication in Vero E6 cells relative to remdesivir (5 µM) (Figure 5A). SBI-0498 or SBI-5844 at 10 µM was as effective as remdesivir (1 µM) in inhibiting OC43 in A549 cells, which were infected at 50% efficiency, compared with the Vero E6 cells (Figure 5A). Principal component analysis (PCA) of both lines revealed distinct clustering patterns in OC43-infected compared to uninfected control cells and infected and treated samples, suggesting significant differences in overall gene expression (Figure 5B and Supplementary Figure S4B). Notably, as clearly noted in segregation of samples along the main principal component (PC1), which accounts for 42.9% of variance in the data, exposure of Vero E6 cells to either SBI-5844 or SBI-0498 effectively reverted clustering patterns towards those of non-infected cells, consistent with inhibition of OC43 RNA and protein levels (Figure 5B). Differential expression comparison of OC43-infected *versus* uninfected cells identified significant transcriptional changes in Vero E6 (1874 genes, Figure 5C, Supplementary Table S1) and A549 (1108 genes, Supplementary Table S2) cells. Comparison of genes differentially expressed in OC43-infected *versus* uninfected Vero E6 and A549 cells to data published for SARS-CoV-2 infected Vero E6 (Riva et al., 2020) and ACE2-expressing A549 cells (Blanco-Melo et al., 2020) revealed significant correlation (Pearson correlation test, *p*-value < 2.2e-16) between gene lists identified for OC43- and SARS-CoV-2-infected cells (Pearson correlation coefficients, Vero E6 = 0.66, A549 = 0.62) (Figure 5D, Supplementary Figure S4C). Effectiveness of SBI-5844 and SBI-0498 treatment was also reflected in pathway analysis (using IPA) of differentially expressed genes following viral infection of both A549 and Vero E6 cells: both compounds rescued gene expression to patterns seen in uninfected cells (Figure 5E). These patterns were confirmed in heatmaps for genes expressed in select pathways (Figure 5F, Supplementary Figure S4D). Importantly, results of RNA-seq analyses in Vero E6 cells were validated on a selected subset of genes using qPCR (Supplementary Figure S4E).

## Discussion

There is significant interest in identifying drugs that attenuate coronavirus infection or replication cycles. These efforts range from targeting proteins required for either viral entry into cells or replication, to blocking subsequent pathophysiological changes in cytokine levels or complement system components (Shoaib et al., 2021). While any antiviral drug should selectively limit viral replication with limited impact on host cells, prophylactic treatments are often used to decrease viral load but may lack specificity or have limited effectiveness, as demonstrated for agents used to counter influenza infection (American Academy of Pediatrics Committee on Infectious Diseases, 2007). Of approaches to coronavirus infection, the greatest effort has been devoted to repurposing existing drugs, such as remdesivir, which was originally developed against Ebola virus (Liang et al., 2020). Repurposed drugs, including the eIF4A inhibitor silvestrol (Biedenkopf et al., 2017), have also been tested to target the translational machinery in infected cells. Targeting that machinery is a desirable strategy against several pathologies, including neurodegenerative disease and cancer, given that translation of oncogenes and other disease-causing mRNAs is enhanced in these conditions relative to translation of house-keeping cellular proteins. The same principle applies to RNA viruses, including coronaviruses, given their dependency on the host translational apparatus and, in particular on eIF4F (Xu et al., 2019). Notably, targeting the translation initiation complex may also attenuate the production of effector proteins that drive and orchestrate the anti-viral immune response, independent of the inhibition of coronavirus proteins. Accordingly, targeting the translation machinery should be carefully tuned to minimize potentially negative systemic effects. Given that viral genes are usually subject to the translation that exceeds the rate seen for housekeeping genes, modulating the eIF4F complex levels may provide a sufficient window to allow host protein synthesis while selectively inhibiting the synthesis of viral proteins that are synthesized in a cap-dependent manner, including those of coronaviruses.

Here, we identified novel small molecules to target eIF4F complex components in the context of coronavirus infection. Our initial *in vitro* screen was conducted *in vitro*, using luciferase as a readout for CAP- dependent and -independent activity. That analysis identified 18 compounds, of which 17 were assessed for effects on OC43 RNA and protein production, and cell toxicity. Only one of these, SBI-1232, potently inhibited OC43 transcripts and proteins in both Vero E6 and A549 cells, with minimal effects on cell viability. A positive control for our initial analyses was SBI-756, a small molecule eIF4F inhibitor that primarily targets eIF4G1 and is reportedly effective against several cancers (Feng et al., 2015; Herzog et al., 2021). However, given the toxicity of SBI-756, we performed additional assessments of less toxic SBI-1232 and of structurally similar compounds that show enhanced potency for inhibition of OC43 RNA and protein production. We show that SBI-5844 and SBI-0498 were superior to SBI-1232 in inhibiting OC43 transcripts and proteins with minimal toxicity. Structurally, SBI-5844 and SBI-0498 exhibit three distinct differences from SBI-1232: both compounds replace the hydrolyzable methyl ester at C-3 of the indole, with a methyl ketone, and SBI-5844 has an alkyl substituent at C-7 of the indole, and a methyl group at the ortho position of the pendant phenyl ring. We used remdesivir as point of comparison with our inhibitors given its effectiveness *in vitro*. Notably, we found that compounds analyzed could be superior to remdesivir, based on their highly effective inhibition of OC43 in Vero E6 cells, which are highly susceptible to coronavirus infection and replication. Unlike remdesivir, which blocks viral RNA polymerase activity, both SBI-5844 and SBI-0498 target eIF4F and thus represent distinct approaches to interfering with coronavirus RNA and protein levels. Notably, while in living cells SBI-0498 inhibits eIF4E:eIF4G binding without triggering dissociation of either from mRNA, SBI-5844-treatment of cells appeared to dissociate eIF4E from the 5′cap. However, both SBI-5844 and SBI-0498 are effective in antagonizing early synthesis of OC43 proteins and in blocking concomitant viral transcripts and proteins. Their effectiveness is reflected by PCA analysis, which revealed that both compounds can revert gene expression seen in infected cells to that seen in uninfected cells. Notably, in Vero E6 cells, these effects appear to be more potent than those observed following remdesivir treatment. Moreover, SBI-5844 or SBI-0498 treatment rescued select gene expression patterns seen in OC43-infected cells, including those associated with interferon and cytokine signaling, to the patterns seen prior to infection. It is important to note that while independent methods were used to monitor the effective inhibition of coronavirus, at either 24 or 48 h post-infection, future studies will extend these measurements to assess the effectiveness of these compounds at later time points post-infection, which will also allow the assessment of viral supernatants and determine effectiveness on viral replication.

Recognizing that OC43 coronavirus strain exhibit different kinetics and cytotoxicity as compared with the SARS-COV2, our study offers mechanistic insight into the pathway that is commonly shared between the two coronaviral strains–the dependency on the requirement of the eIF4F complex for the productive viral life cycle. It is therefore expected that effective targeting of the eIF4F complex may offer a novel strategy for impeding the life cycle of coronaviruses. Our study’s relevance to SARS-CoV-2 comes is supported by the finding that gene expression changes mapped following OC43 infection overlap with those seen following SARS-CoV-2 replication, and, correspondingly, that gene expression changes seen following SARS-CoV-2-infection of ACE-2-expressing A549 cells but blocked by remdesivir treatment also overlapped with those seen in SBI-5844 or SBI-0498-treated OC43-infected A549 cells. Yet, precise mechanism for the mode of these compounds effect on viral transcripts and proteins requires further studies, given that protein level was largely attenuated without changes in translational efficiencies of corresponding mRNAs.

Notably, while effective in culture-based systems, as demonstrated here for Vero E6 cells infected with OC43, the biophysical properties of both SBI-0498 and SBI-5844 need improvement, given their rapid clearance and limited solubility.

Overall, targeting the eIF4F complex offers important advantages over other cellular targets currently being investigated for coronavirus inhibition. Even though these viruses will acquire many mutations, they will require the host translation machinery to support their life cycles. Both novel small molecule eIF4F complex inhibitors identified here effectively inhibit OC43 protein and RNA levels in cells with minimal toxicity. Furthermore, at least in cell line models, these compounds’ effectiveness appears superior to that of remdesivir, based on superior inhibition in cells with high infection rate (i.e., Vero E6) and concomitant reversion of gene expression patterns to those of uninfected cells. Further optimization and development of these compounds are highly warranted, as they offer a new therapeutic modality against rapidly expanding coronaviruses.

## Methods

### Cell culture

A549, Vero E6, HeLa-ACE2, and HCT-8 cells were obtained from the American Type Culture Collection (ATCC). A549 and Vero E6 cells were maintained in high-glucose Dulbecco’s modified Eagle’s medium (HyClone) with 10% fetal bovine serum and 1% penicillin-streptomycin at 37°C in 5% CO_2_. HCT-8 cells were cultured in RPMI-1640 medium (HyClone) with 10% fetal bovine serum and 1% penicillin-streptomycin at 37°C in 5% CO_2_.

### Reagents

Compounds used in this study were purchased from ChemBridge, MolPort or VitaScreen, as indicated. Physical properties were calculated using Marvin (Ver. 21.17.0) ChemAxon, www.chemaxon.com). Remdesivir was obtained from Cayman Chemical Company. Compounds were dissolved in DMSO and stored as a 10 mM stock solution at −20°C. Each compound was freshly prepared (from the stock solution) before use.

### Virus propagation

OC43 virus was obtained from ATCC and propagated according to ATCC protocols. Briefly, OC43 virus was propagated in HCT-8 cells at 90% confluency (18–48 h-old monolayers). Monolayers were washed twice with PBS or serum free medium prior to inoculation. Virus was diluted in a minimum volume with serum free medium, and cells were infected at multiplicity of infection (MOIs) of 0.01–0.1. Cells were incubated with virus 1–2 h at 33°C in humidified 5% CO_2_ with continuous rocking. The adsorption phase was ended by addition of growth medium (RPMI supplemented with 2% FBS), and culture continued at 33°C in a humidified 5% CO_2_ incubator. Supernatants were harvested 4–6 days later, filtered using a 0.45 µm filter, and stored as aliquots at −80°C until use. Virus was propagated in Vero E6 (ATCC^®^ CRL-1586™) cells and stored at −80°C in aliquots.

### Virus titration

HCT-8 cells were seeded in 96-well plates (100 µL/well) until confluent. Serial 10-fold dilutions of OC43 stock were prepared using RPMI supplemented with 2% FBS and then transferred to corresponding wells. Plated cells were cultured 4 days at 33°C in a humidified atmosphere of 5% CO_2_. Cytopathic effects on infected cells were quantified using crystal violet staining.

### Virus infection

Cells were seeded in 6-well (2 x 105) or 96-well (1 x 10^4^) plates. The next day, cells were incubated 1 h with a dilution of OC43 virus stock at a MOI of 0.1. Media supplemented with 2% FBS and test compounds at various concentrations were then added to infected cells. Cells were collected for Western blotting analysis and RNA-seq (6-well plate) after 24 h or subjected to IF staining (96-well plate) after 48 h.

### Western blot analysis and antibodies

Cells were rinsed with PBS and lysed as previously described (Feng et al., 2015). Protein concentration was determined using Coomassie Plus Protein Assay Reagent (Thermo Scientific). Equal amounts of cell lysate proteins (50 μg) were separated on SDS-PAGE and transferred to polyvinylidene difluoride membranes (PerkinElmer Life Sciences). Membranes were blocked in 3% BSA/TBST for 1 h and incubated with primary antibodies (overnight at 4°C), with shaking. Following three TBST washes, membranes were incubated for 1 h at room temperature with secondary antibodies (1:10,000). Detection and quantifications were made using an Odyssey Infrared Imaging System (LICOR Biosciences). Antibodies to OC43 N-protein and the spike protein were obtained from EMD Millipore (cat# MAB9012) and CUSABIO (cat# CSB-PA336163EA01HIY), respectively. Antibodies against eIF4G1, eIF4E and 4EBP1 were purchased from Cell Signaling Technology (respective cat# 8701, cat# 2067, and cat# 9644), and HSP90 antibody was obtained from Santa Cruz Biotechnology (cat# sc-13119). Secondary antibodies were goat anti-rabbit Alexa Fluor 680 (cat# A21076) and goat anti-mouse Alexa Fluor 680 (cat# A21057) (Invitrogen). All antibodies were used according to suppliers’ recommendations.

### Cell viability assay

Cell viability assay was performed in uninfected Vero E6 and A549 cells. Cells were seeded (8,000 cells in 100 μL per well) in 96-well plates and allowed to attach overnight. Test compounds were serially diluted from stock solutions (10 mM) and added to cells. Assays were performed in triplicate, and each microplate included media and DMSO control wells. Cell viability was assessed 48 h after treatment using CellTiter-Glo (Promega) according to the manufacturer’s protocol. Cell growth inhibition was calculated as a percentage of DMSO-treated controls.

### Immunofluorescence staining for OC43

A549 cells were seeded in 96-well black plates. After 24 h, cells were infected with OC43 and treated with different compounds following the infection. After 48 h, cells were fixed (4% paraformaldehyde for 10 min at room temperature) and washed three times with PBS. Cells were then permeabilized with PBS containing 0.3% Triton X-100 for 10 min and blocked with 3% BSA for 1 h. Primary antibody against OC43 N-protein (EMD, 1:1000) was then added and cells were incubated at 4°C overnight. Cells were washed three times with PBS containing 0.1% Triton X-100 followed by incubation with Alexa Fluor 488 goat anti-mouse secondary antibody (Invitrogen, 1:500) for 1 h at room temperature. After washing three times with PBS containing 0.1% Triton X-100, cells were stained with DAPI for 15 min before imaging using a fluorescence microscope. Infected cells (green, OC43 N-protein staining followed by Alexa Fluor 488 staining) and total (DAPI staining) cells were quantified using the Celigo imaging cytometer.

### Fluorescence correlation spectroscopy (FCS)

FCS trajectories were obtained using a Leica SP8 Falcon microscope equipped with a Leica APO 86x/N.A. = 1.20 water-immersion objective with the motCORR automated correction collar, as previously described (Gandin et al., 2021). Mouse embryonic stem cells (mESCs) in which Halo and SNAP_f_ tag were inserted into the EIF4E1 and EIF4G1 alleles, respectively, were differentiated into fibroblasts (Gandin et al., 2021). Twenty-four hours later, cells were labelled with 100 nM JF585-Halo tag ligand for 10 min and 500 nM JF64-6SNAP_f_ tag ligand for 45 min. A set of two measurements (10 s each) were performed in the cytoplasm and in the nucleus for the indicated conditions in at least 5 cells per condition. FCS and FCCS values were exported and averaged using GraphPad Prism v7.

### m^7^GTP pull-down assay

As previously described (Feng et al., 2015), cells growing in 100 mm plates were washed with cold PBS, collected, and lysed in lysis buffer [50 mM MOPS/KOH (pH 7.4), 100 mM NaCl, 50 mM NaF, 2 mM EDTA, 2 mM EGTA, 1% NP40, 1% Na-DOC, 7 mM β-mercaptoethanol (β-ME), plus protease inhibitors and a phosphatase inhibitor cocktail). Lysates were incubated with m^7^-GDP-agarose beads (Jena Bioscience) for 20 min with rotation and washed three times with wash buffer [50 mM MOPS/KOH (pH 7.4), 100 mM NaCl, 50 mM NaF, 0.5 mM EDTA, 0.5 mM EGTA, 7 mM β-ME, 0.5 mM PMSF, 1 mM Na_3_VO_4_ and 0.1 mM GTP]. Bound proteins were eluted by boiling beads in loading buffer. Material pulled down by m^7^-GDP-agarose was analyzed by Western blot.

### Reverse transcription and real-time qPCR

Total RNA was extracted using a total RNA miniprep kit (Sigma) with the On-column DNase I digestion step included. cDNA was synthesized using a cDNA kit from Applied Biosystems according to the manufacturer’s protocol. Real-time PCR was performed on a Bio-Rad CFX Connect Real-Time System using FastStart universal SYBR Green Master from Bio-Rad. H3A was used as an internal control. Quantitative PCR reactions were performed in triplicate. PCR primers were designed using PrimerBank (http://pga.mgh.harvard.edu/primerbank). Primers are listed in Supplementary Table S3.

### Pulse chase ^35^S-labeling

Vero E6 cells were seeded and cultured overnight followed by infection with OC43. Twenty-four hours later, cells were washed two times with phosphate buffered saline (PBS) and covered with 2 ml of DMEM without methionine and cysteine supplemented with 2% dialyzed FBS. After 60 min, a^35^S-methionine and ^35^S-cysteine mixture was added (50 μCi/ml, PerkinElmer, United States) along with SBI-584, SBI-0498 or remdesivir (10 µM). Cells were incubated 4 h and excess radioactive material was removed. Cells were then washed several times with PBS and subjected to protein extraction. Equal amounts of radioactive labeled proteins were used to perform immunoprecipitation using antibodies against the OC43 N-protein. Immunoprecipitated material was washed 3 times with PBS before they were separated by SDS-PAGE, which was electro-transferred to a PVDF membrane. Membranes were treated with EN^3^HANCE to enhance the ^35^S signal before exposure to X-ray film. Autoradiograms were quantified using ImageJ.

### Polysome profiling

Vero E6 cells were seeded in 15-cm plates, infected with OC43 and treated with 10 μM of SBI-5844 and SBI-0498. After 12 h, cells were harvested and lysed in hypotonic lysis buffer (5 mM Tris HCl, pH 7.5, 2.5 mM MgCl_2_, 1.5 mM KCl, 100 μg/ml cycloheximide, 2 mM DTT, 0.5% Triton, 0.5% sodium deoxycholate). Polysome-profiling was carried out as described by Gandin et al. (Gandin et al., 2021). Fractions were collected and RNA was extracted using TRIzol according to the manufacturer’s instructions. RT and qPCR were performed as described above. Experiments were done in independent triplicates whereby every sample was analyzed in a technical triplicate.

### RNA-seq

Total RNA was extracted using a total RNA miniprep kit (Sigma) with the On-column DNase I digestion step included. Libraries were prepared from isolated total RNA using the QuantSeq 3′ mRNA-Seq Library Prep Kit FWD for Illumina from Lexogen (Vienna, Austria). Barcoded libraries were pooled, and single end sequenced (1 x 75) on the Illumina NextSeq 500 system using the High output V2.5 kit (Illumina Inc., San Diego, CA). Read data was processed and multiplexed with the BlueBee Genomics Platform (BlueBee, San Mateo, CA).

### RNA-seq data processing

We used Cutadapt v2.3 to trim llumina TruSeq adapter, polyA, and polyT sequences from raw reads with cutadapt v2.3 (https://doi.org/10.14806/ej.17.1.200). Trimmed reads were aligned to human genome version hg38 (A549) or *Chlorocebus sabaeus* genome version ChlSab1.1 (Vero E6) with STAR aligner v2.7.0d_0221 (Dobin et al., 2013) using ENCODE long RNA-seq pipeline (https://github.com/ENCODE-DCC/long-rna-seq-pipeline) parameters. Gene expression levels were quantified using RSEM v1.3.1 (Li et al., 2011). We used Ensembl gene annotations version 84 (A549) or 104 (Vero E6) in alignment and quantification steps. FastQC v0.11.5 (https://www.bioinformatics.babraham.ac.uk/projects/fastqc/) and MultiQC v1.8 (Ewels et al., 2016) were used to assess quality of raw RNA-seq data, alignments, and quantification. Biological replicate concordance was assessed using principal component analysis (PCA) and pair-wise Pearson correlation analysis. Genes expressed at low levels with estimated counts (from RSEM) fewer than five times the number of samples were filtered and discarded from downstream analysis. DE genes were identified using DESeq2 v1.22.2 based on a generalized linear model and negative binomial distribution (Love et al., 2014). Genes with Benjamini–Hochberg corrected *p*-value < 0.05 and fold-change ≥ 2.0 or ≤ −2.0 were identified as DE. Pathway analysis and comparison of DE gene lists were performed using Ingenuity Pathway Analysis (Qiagen, Redwood City, United States).

Differentially up- and down-regulated genes from A549 and Vero E6 OC43 vs. control comparisons were compared to published COVID-19 studies curated in Coronascape (Wang et al., 2021). The top matched datasets were from Blanco-Melo et al. (Blanco-Melo et al., 2020) and Riva et al. (Riva et al., 2020). For comparison to the full list of DE genes, Supplementary Table S1 from Blanco-Melo et al. was downloaded and compared to A549 RNA-seq data in this study. Read counts for Vero E6 SARS-CoV-2 infected *versus* control from the study of Riva et al. were downloaded from GEO (accession number GSE153940) and DE genes identified using DESeq2 as described above. Unmapped reads to human or *Chlorocebus sabaeus* genomes were mapped to the OC43 genome (NCBI accession number AY391777.1) to assess the percentage of viral reads in all samples. RNA-seq main and supplemental figures were plotted using ggplot2, ggpubr, and ComplexHeatmap. RNA-seq main and supplemental figures were plotted using ggplot2 (Dobin et al., 2013). Data is available in GEO, accession number GSE198400.

### *In vitro* translation assay and high throughput screen

A bicistronic dual-reporter construct that harboring the firefly luciferase (FF) sequence followed by an encephalomyocarditis virus (EmCV) IRES and the Renilla reniformis (Ren) luciferase sequence was previously described by Bordeleau *et al.* (Bordeleau et al., 2006) and generously provided by Dr. Jerry Pelletier. The FF-EmCV-Ren construct was linearized with BamHI-HF (NEB, Ipswich, MA, United States), purified by ethanol precipitation and transcribed using the mMessage Machine T7 Ultra Kit based on manufacturer’s instructions. Resulting mRNAs were purified using the MegaClear cleanup kit (Ambion).

To quantify translation from the FF-EmCV-Ren construct, rabbit reticulocyte lysates (RRLs) (Life Technologies) and the Dual-Glo^®^ (Promega) read-out were chosen. Briefly, 2 µL RRL mixed 1:1 with Translation Mix 20x (-Met) and Translation Mix 20x (-Leu) was pre-incubated 1 h with test compounds in white 1536-well plates (#3725, Corning) at room temperature (RT). Translation was initiated by adding 0.1 µg RNA per well followed by incubation for 2 h at 30°C. After a short cooling phase, 2.5 µL Dual-Glo reagent one was added to each well and incubated 10 min before reading FF luciferase activity with the ViewLux™ uHTS Microplate Imager (Perkin Elmer). Immediately after, 2.5 µL Dual-Glo reagent two was dispensed to each well and incubated 10 min at RT before reading Ren luciferase activity on the ViewLux. Cycloheximide (62.5 µM) was used as positive control for translation inhibition and DMSO only (0.5% final) as vehicle control. To exclude the possibility that test compounds interfere with luciferase activities, a counter-assay was performed in which test compounds were added after completion of *in vitro* translation and then read with the ViewLux uHTS Microplate Imager. In this case, any inhibition of FF or Ren luciferase would have been flagged as a false-positive. Pifithrin (20 µM) served as positive control and DMSO only (0.5% final) as vehicle control. All test, control, and benchmark compounds were acoustically dispensed using an ECHO^®^ 555 liquid handler (Labcyte), and reaction reagents were transferred using a BioRAPTR^®^ microfluidic dispenser (Beckman Coulter, Brea, CA). Plates were handled by an HTS lab automation platform designed by HighRes Biosolutions (Berverly, MA). HTS data was corrected and normalized by Screener^®^ (Genedata, Basel, Switzerland). Assay data were then transferred and analyzed using CBIS (ChemInnovation Software, San Diego). IC_50_ values were calculated using a 4-parameter fit.

### Compound library

We used a large library of approximately 320,000 compounds selected from a pool of over three million compounds from five chemical vendors (ChemBridge, Asinex, Enamine, Life Chemicals, and ChemDiv). Compounds were selected for general HTS screening using cheminformatics selection strategies, such as a cluster-based 2D-fingerprint approach, to ensure diversity balanced with good representation and good physicochemical properties. Appropriate filters were applied to exclude compounds with unwanted or reactive groups. Natural products, compounds from kinase-focused libraries, an FDA-approved drug collection, and selected fragments were also included, for a total of 338,000 compounds screened in this HTS campaign.

### Cytotoxicity counter-screen

To exclude compounds with off-target effects, we performed a cytotoxicity counter-screen using the human colorectal carcinoma line HCT116 (ATCC^®^ CCL-247™). 200 cells/well in 5 µL medium were seeded into tissue culture-treated 1536-well plates (#3893, Corning), covered with MicroClime^®^ Lids (Labcyte) and incubated at 37°C in 5% CO_2_ overnight. The next day, test compounds at a final concentration of 10 µM were added. Staurosporine (final concentration 0.5 µM) served as positive control, while DMSO only (0.5% final concentration) served as vehicle control. All 1586-well plates were then incubated at 37°C in 5% CO_2_ for 72 h. Then, 4 µL CellTiter-Glo^®^ (Promega) was added to each well and samples were read with a PHERAStar^®^ FS microplate reader (BMG Labtech).

## Statistics and reproducibility

Statistical significance was assessed by ordinary one-way ANOVA. GraphPad Prism nine software (Graphpad, La Jolla, CA) was used for all statistical calculations. All cell culture experiments were performed three times. Data is presented as mean ± SD (unless noted otherwise in figure legends). A *p*-value less than 0.05 was considered statistically significant.

## Data Availability

The datasets presented in this study can be found in online repositories. The names of the repository/repositories and accession number(s) can be found in the article/Supplementary Material.
