# Corrigendum: Inhibition of coronavirus HCoV-OC43 by targeting the eIF4F complex

**DOI:** 10.3389/fphar.2024.1528449

**Published:** 2024-12-19

**Authors:** Yongmei Feng, Stefan Grotegut, Predrag Jovanovic, Valentina Gandin, Steven H. Olson, Rabi Murad, Anne Beall, Sharon Colayco, Paul De-Jesus, Sumit Chanda, Brian P. English, Robert H. Singer, Michael Jackson, Ivan Topisirovic, Ze’ev A. Ronai

**Affiliations:** ^1^ Cancer Center at Sanford Burnham Prebys Medical Discovery Institute, La Jolla, CA, United States; ^2^ Conrad Prebys Center for Chemical Genomics at Sanford Burnham Prebys Medical Discovery Institute, La Jolla, CA, United States; ^3^ Lady Davis Institute, SMBD Jewish General Hospital, Gerald Bronfman Department of Oncology and Division of Experimental Medicine, McGill University, Montreal, QC, Canada; ^4^ Janelia Research Campus, Howard Hughes Medical Institute, Ashburn, VA, United States; ^5^ Immunology and Infectious Disease Center at Sanford Burnham Prebys Medical Discovery Institute, La Jolla, CA, United States

**Keywords:** COVID-19, OC43, SARS-CoV-2, coronavirus, eIF4F, translation initiation complex, Vero E6, A549

In the published article, there was an error in Figure 4 as published. One of the lanes (referring to eIF4E) was mistakenly replaced with a control lane (third panel from the top in Figure 4A). The corrected Figure 4 and its caption appear below.

**FIGURE 4:**
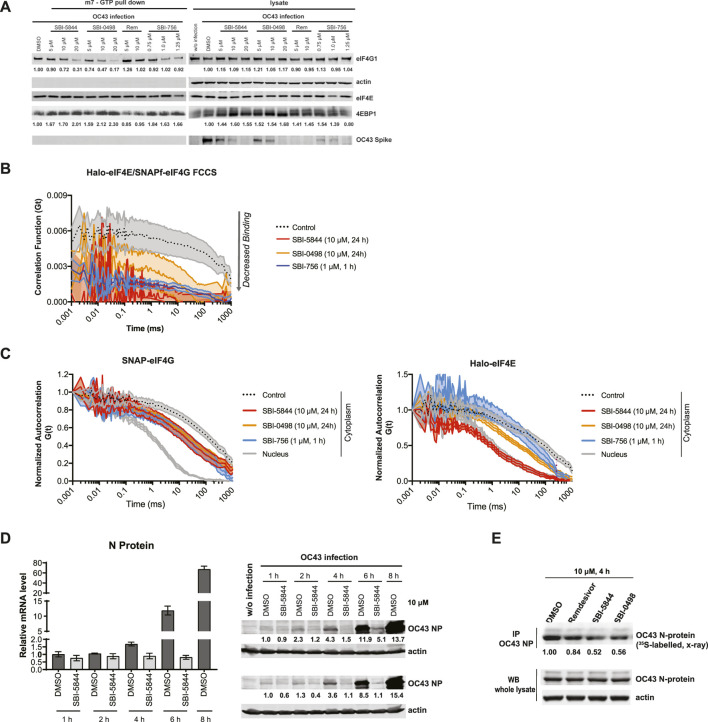
SBI-5844 and SBI-0498 attenuates translation initiation complex function. **(A)** Western blot analysis of indicated proteins prepared from A549 cells infected with OC43 and treated with indicated compounds. Cell lysates were incubated with m7GTP-agarose beads to capture the eIF4F complex. Shown is western blot of both total cell lysates (right panel) and m7GTP-agarose bound proteins (left panel) immunoblotted with indicated antibodies. Each protein band was quantified by ImageJ, normalized to actin (right panel) or eIF4E (left panel) as indicated. **(B)** Simultaneous diffusion of endogenous JF585Halo-eIF4E and JF646SNAPf-eIF4G1 throughout the focal volume was analyzed in living cells by dual color cross-correlation spectroscopy in the indicated conditions (Mean ± SEM; N = 10). Cross-correlation, due to eIF4F:eIF4G interaction, was detected in the cytoplasm of cells treated with vehicle control. Minimal cross-correlation was detected upon treatment with SBI-756, SBI-5844 or SBI-0498. **(C)** Diffusion of endogenous JF646SNAPf-eIF4G1 (left panel) and JF585Halo-eIF4E (right panel) was measured by fluorescence correlation spectroscopy in living cells treated with vehicle control or indicated compounds, in the indicated cellular compartment (Mean ± SEM; N = 10). Nuclear temporal autocorrelation depicts diffusion of free-diffusing JF646SNAPf-eIF4G1 (upper panel) and JF585Halo-eIF4E (lower panel). The autocorrelation curves were normalized for visual comparison of shape. **(D)** Vero E6 cells were infected with OC43 and treated with SBI-5844 for indicated times. RNA and protein were extracted and subjected to RT-qPCR analysis for virus RNA (left, mean ± SD, n = 3) and WB for virus N-protein (right). Each protein band was quantified by ImageJ, normalized to actin levels as indicated in numbers shown under the blots. **(E)** Vero E6 cells were infected with OC43 for 24 h. Cells were then treated with indicated compounds and radiolabeled with 35S-methionineand and 35S-cysteine for newly synthesized protein for 4 h. OC43 N-protein was immunoprecipitated (IP), separated by SDS-PAGE, transferred to PVDF membrane, and exposed to x-ray film. Each band was quantified by ImageJ, normalized to OC43 N-protein level as indicated under the blots.

The authors apologize for this error and state that this does not change the scientific conclusions of the article in any way. The original article has been updated.

