# Supplementary figures and images for: Inhibition of coronavirus HCoV-OC43 by targeting the eIF4F complex
Source: Front Pharmacol. 2022 Dec 1;13:1029093. doi: 10.3389/fphar.2022.1029093 (PMC9751428; doi:10.3389/fphar.2022.1029093)

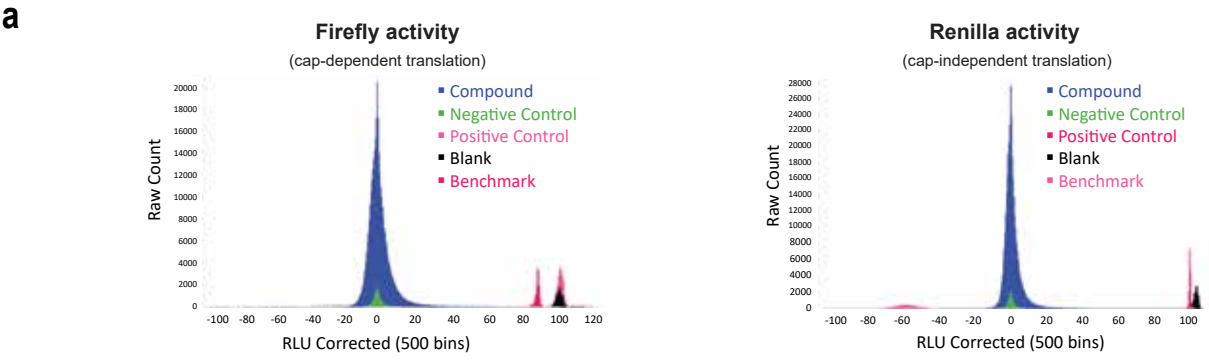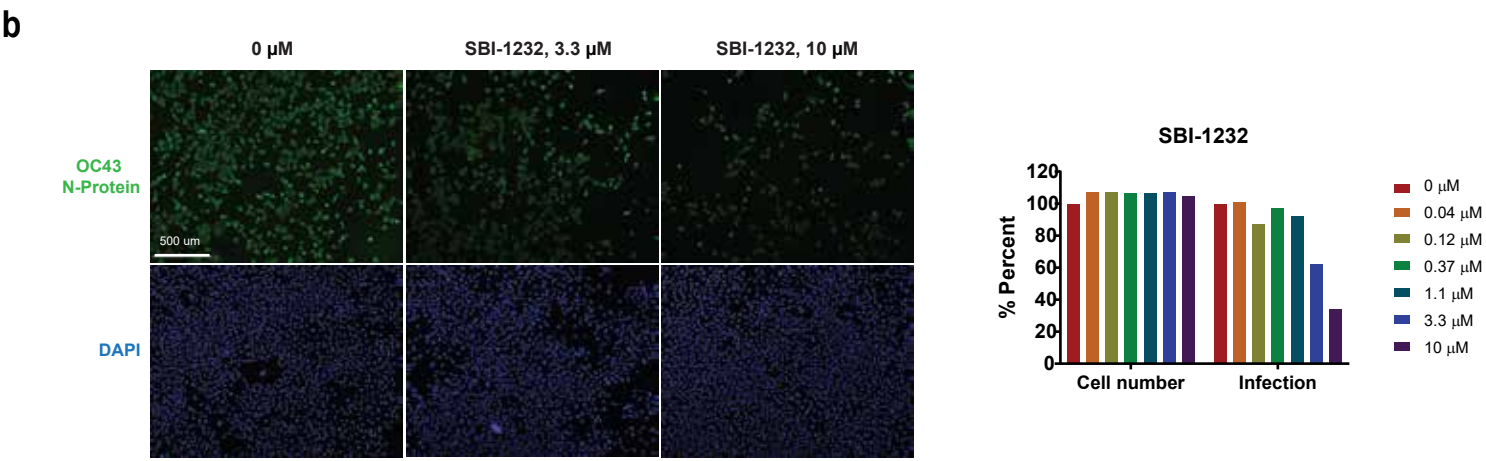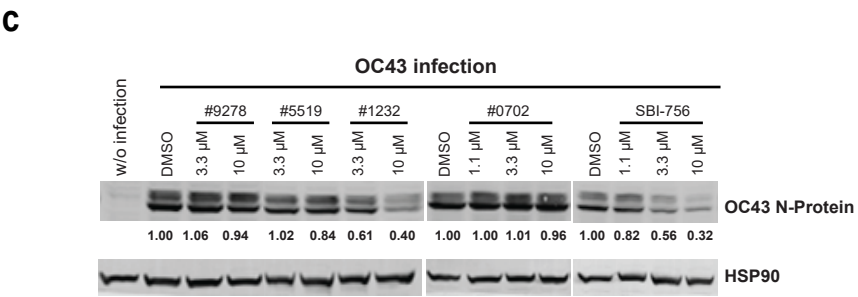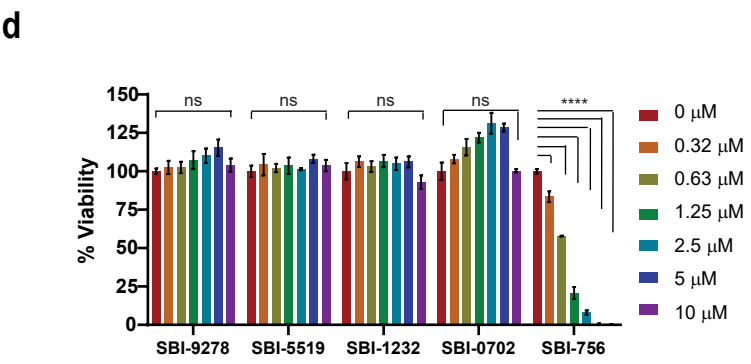

**a**

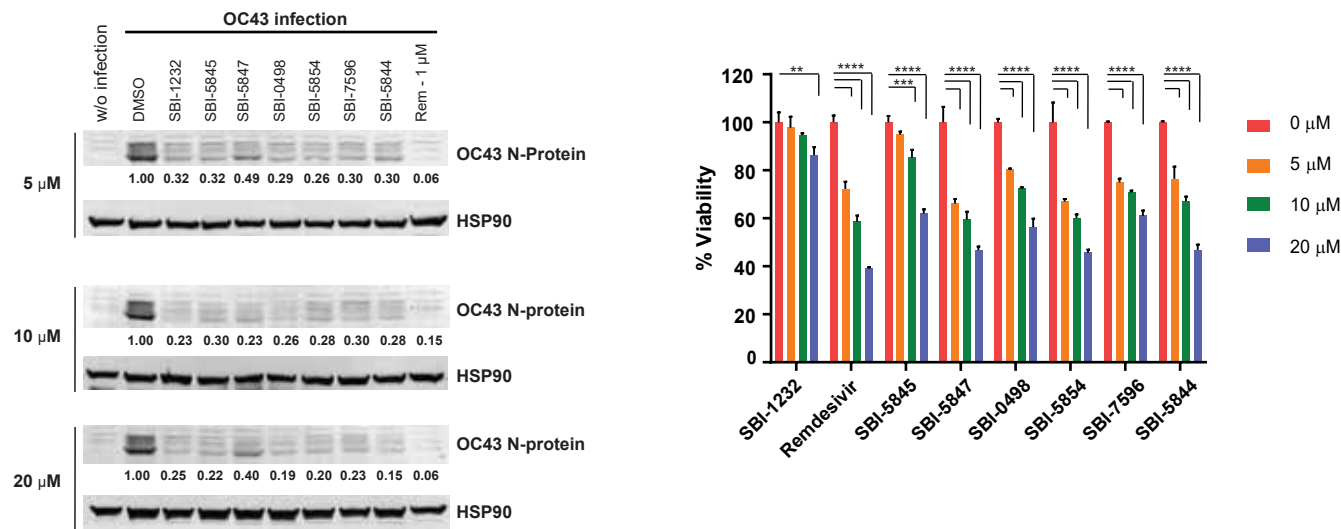

**b**

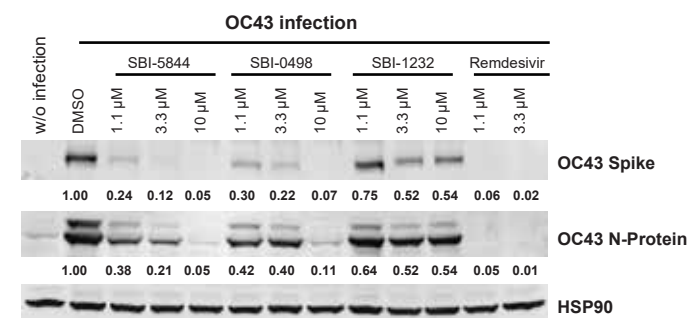

**c**

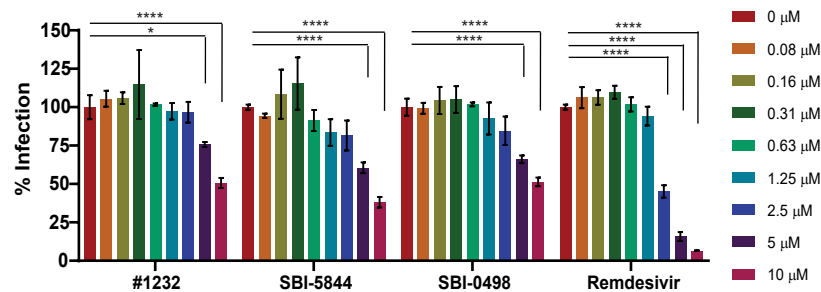

**d**

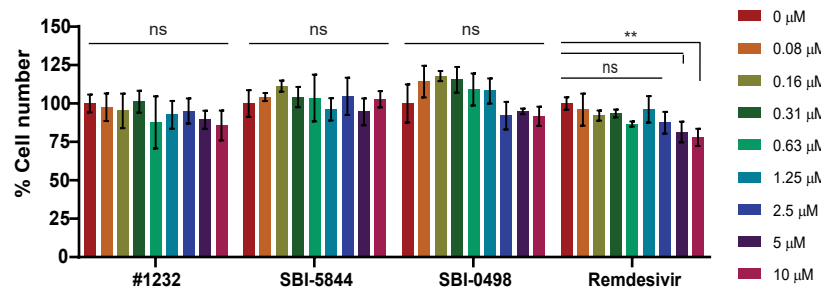

**e**

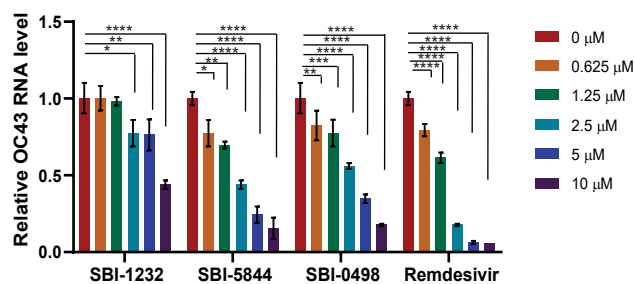

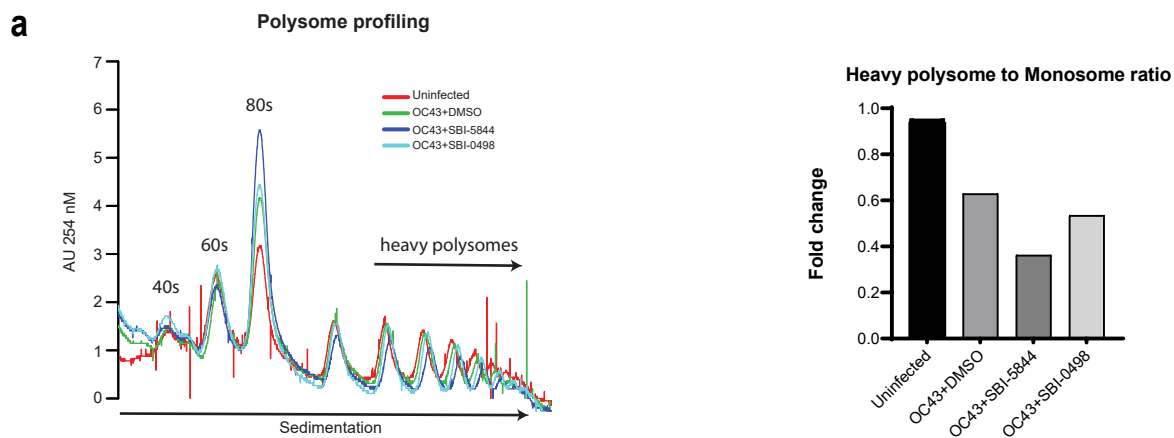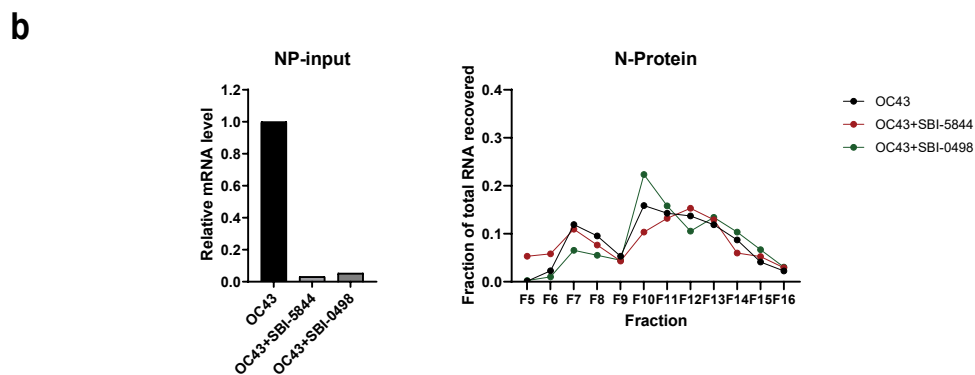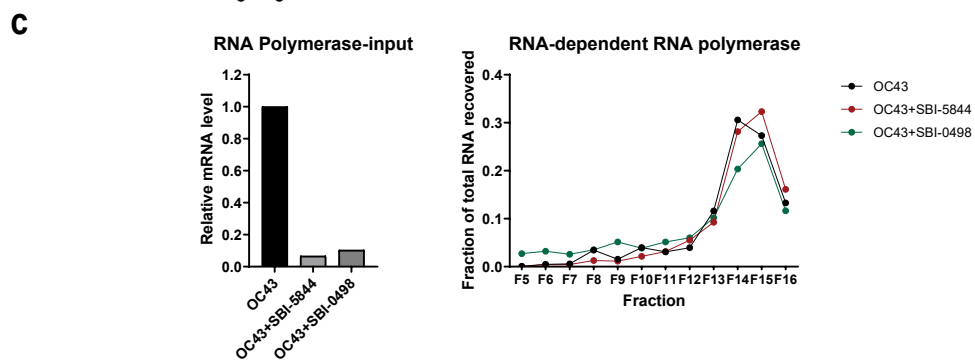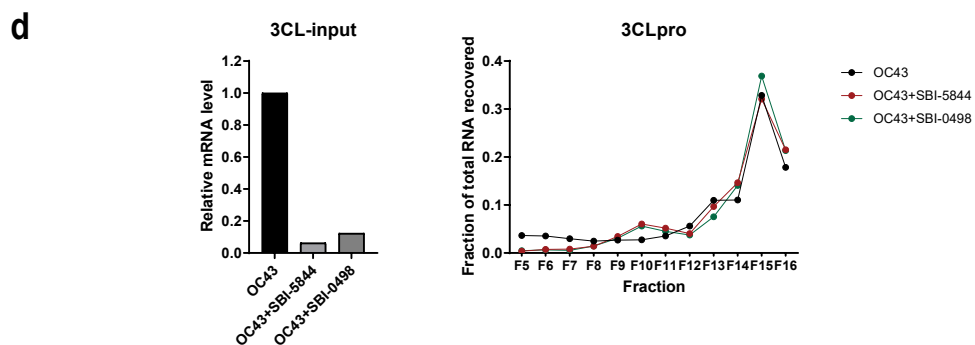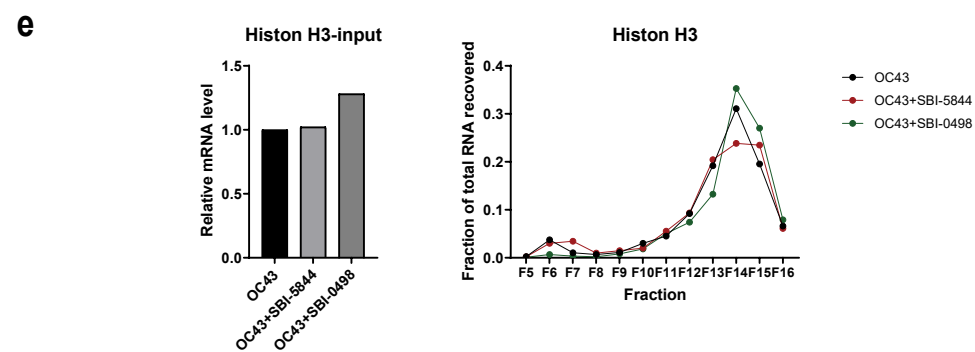

a

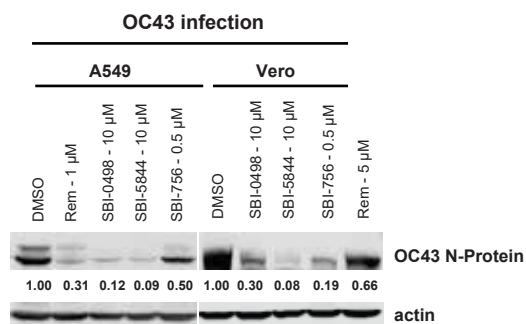

b

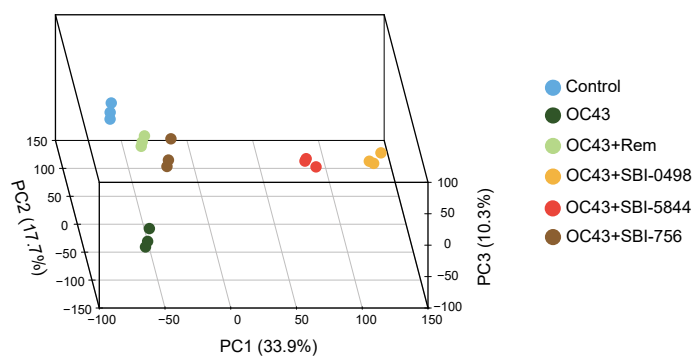

c

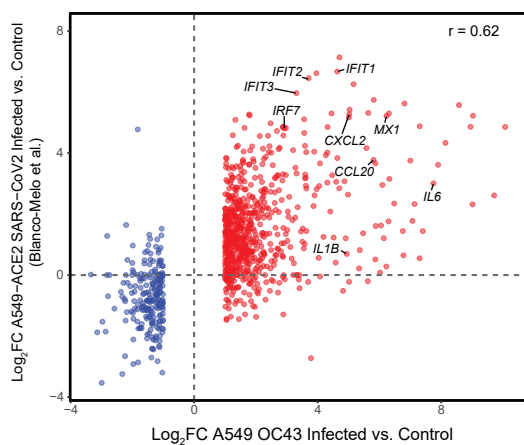

d

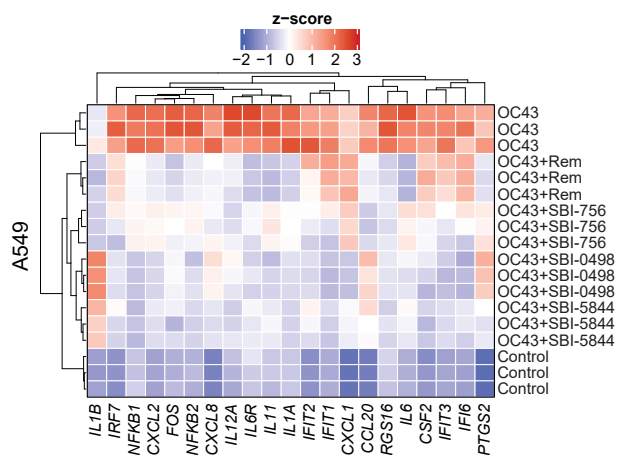

e

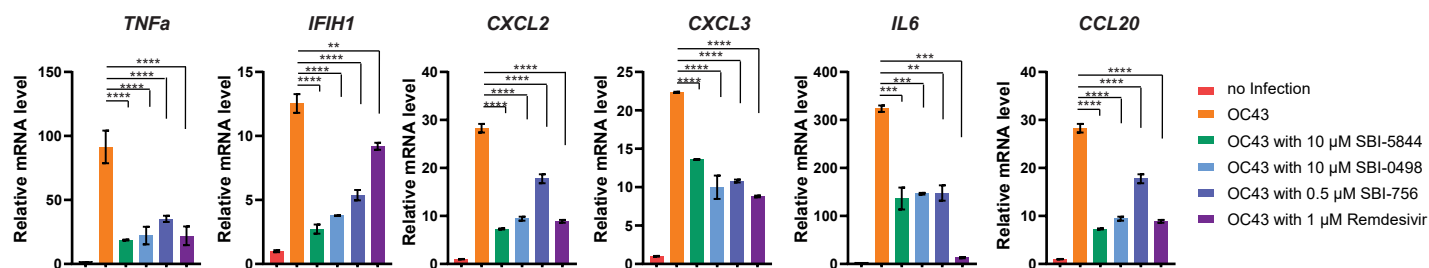

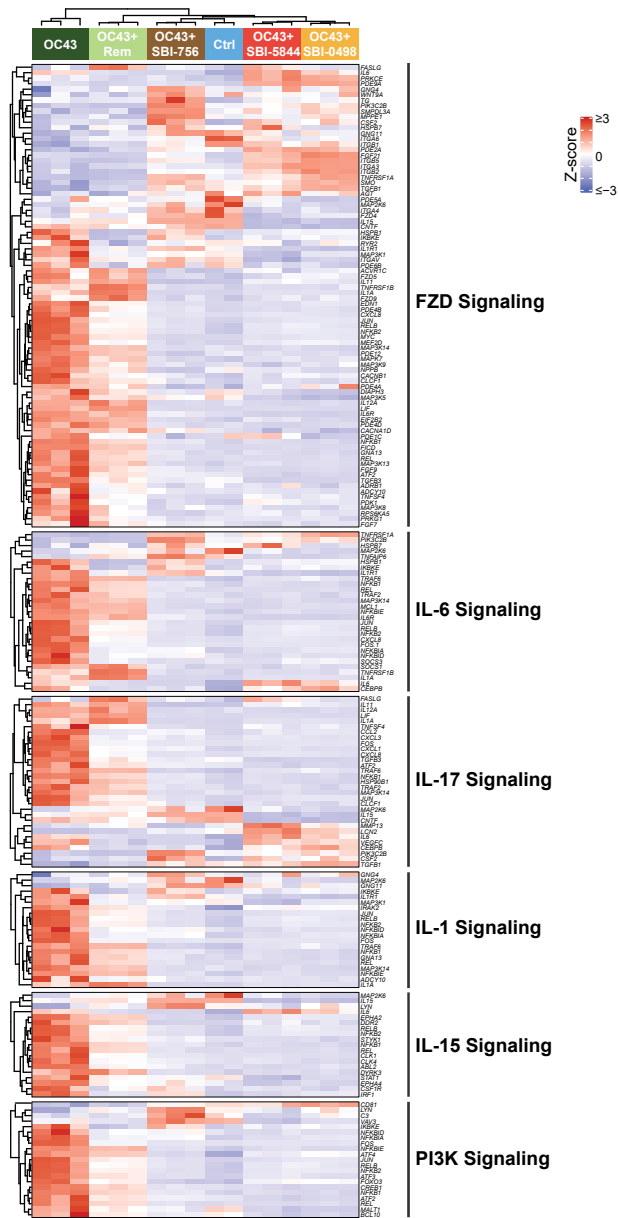

Supplement: Supplementary file 1 [file Presentation1.pdf]
